# Stratification of patients with clear cell renal cell carcinoma to facilitate drug repositioning

**DOI:** 10.1016/j.isci.2021.102722

**Published:** 2021-06-12

**Authors:** Xiangyu Li, Woonghee Kim, Kajetan Juszczak, Muhammad Arif, Yusuke Sato, Haruki Kume, Seishi Ogawa, Hasan Turkez, Jan Boren, Jens Nielsen, Mathias Uhlen, Cheng Zhang, Adil Mardinoglu

**Affiliations:** 1 Science for Life Laboratory, KTH—Royal Institute of Technology, Stockholm 17165, Sweden; 2 Bash Biotech Inc, 600 West Broadway, Suite 700, San Diego, CA 92101, USA; 3 Department of Pathology and Tumor Biology, Institute for the Advanced Study of Human Biology (WPI-ASHBi), Kyoto University, Kyoto 606-8501, Japan; 4 Department of Urology, Graduate School of Medicine, The University of Tokyo, Tokyo 113-8654, Japan; 5 Centre for Hematology and Regenerative Medicine, Department of Medicine, Karolinska Institute, Stockholm 17177, Sweden; 6 Department of Medical Biology, Faculty of Medicine, Atatürk University, Erzurum 25240, Turkey; 7 Department of Molecular and Clinical Medicine, University of Gothenburg, Sahlgrenska University Hospital, Gothenburg 41345, Sweden; 8 Department of Biology and Biological Engineering, Chalmers University of Technology, Gothenburg 41296, Sweden; 9 BioInnovation Institute, Copenhagen N 2200, Denmark; 10 Key Laboratory of Advanced Drug Preparation Technologies, School of Pharmaceutical Sciences, Ministry of Education, Zhengzhou University, Zhengzhou 450001, China; 11 Centre for Host-Microbiome Interactions, Faculty of Dentistry, Oral & Craniofacial Sciences, King's College London, London SE1 9RT, UK

**Keywords:** bioinformatics, systems biology, cancer systems biology, omics

## Abstract

Clear cell renal cell carcinoma (ccRCC) is the most common histological type of kidney cancer and has high heterogeneity. Stratification of ccRCC is important since distinct subtypes differ in prognosis and treatment. Here, we applied a systems biology approach to stratify ccRCC into three molecular subtypes with different mRNA expression patterns and prognosis of patients. Further, we developed a set of biomarkers that could robustly classify the patients into each of the three subtypes and predict the prognosis of patients. Then, we reconstructed subtype-specific metabolic models and performed essential gene analysis to identify the potential drug targets. We identified four drug targets, including *SOAT1*, *CRLS1, and ACACB,* essential in all the three subtypes and *GPD2*, exclusively essential to subtype 1. Finally, we repositioned mitotane, an FDA-approved *SOAT1* inhibitor, to treat ccRCC and showed that it decreased tumor cell viability and inhibited tumor cell growth based on *in vitro* experiments.

## Highlights

• Three consistent molecular ccRCC subtypes were found to guide patients' prognoses
• REOs-based biomarker was developed to robustly classify patients at individual level
• *SOAT1* is identified as a common drug target for all ccRCC subtypes
• Mitotane was repositioned treatment of ccRCC via inhibiting *SOAT1*

## Graphical abstract

**Figure:**
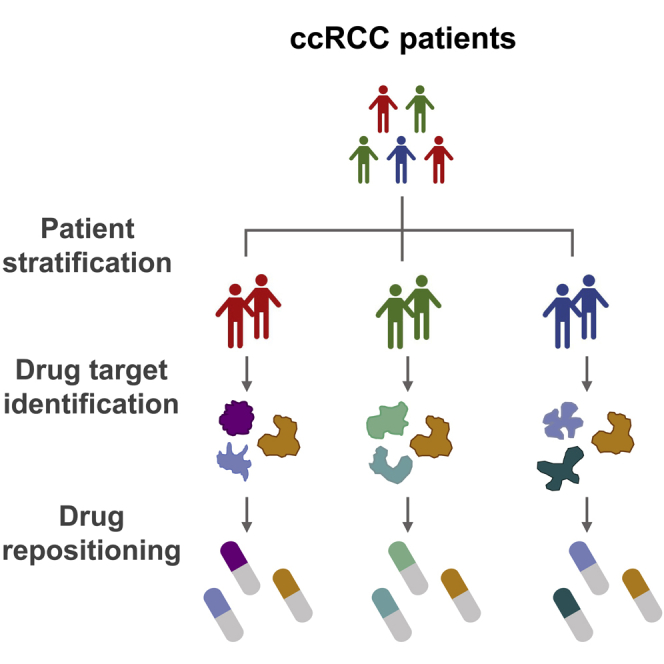

## Introduction

Kidney cancer constitutes more than 3% of all adult malignancies, with 400,000 new cases and 175,000 deaths in 2018 (Bray et al., 2018). Approximately 85% of all kidney tumors are renal cell carcinoma (RCC), and 70% of RCC cases are of clear cell histology (Motzer et al., 2020). Smoking, obesity, and hypertension are established risk factors for RCC development (Motzer et al., 2020). Partial/radical nephrectomy is the standard primary treatment for localized tumors. However, 30–35% of patients relapse or distant metastases after nephrectomy, and these patients need further adjuvant chemotherapy (Porta et al., 2019). The most frequently occurring genetic event in clear cell renal cell carcinoma (ccRCC) is the deletion or inactivated mutation of the gene von Hippel-Linadau (*VHL*) tumor suppressor, which is involved in the ubiquitination and degradation of hypoxia-inducible factors (*HIF-1α* and *HIF-2α*) (Cancer Genome Atlas Research, 2013; Masson and Ratcliffe, 2014). Accumulated hypoxia-inducible factors promote tumor cells to develop an adaptive response to hypoxic stress through transcriptional activation of genes related to glucose metabolism, cell proliferation, migration, and angiogenesis (Masoud and Li, 2015). Recently, clinically recommended target therapies that modulate the downstream pathways after *HIF* activation include tyrosine kinase inhibitors, such as sunitinib, axitinib, sorafenib, pazopanib, cabozantiniband lenvatinib, and mTOR inhibitors, such as everolimus and tesirolimus, and anti-VEGF antibodies such as bevacizumab (Figlin et al., 2018). However, only 16.6–58% of patients with ccRCC could be attributed to genetic *VHL* alteration (Cowey and Rathmell, 2009). In addition, these drugs' response rates are only 9–40% in different clinical trials (Motzer et al., 2020). Owing to the high inter-tumor or intra-tumor heterogeneity, it is well recognized that various tumors could be driven by different oncogenic pathways, which has a significant effect on the identification of drug targets to guide clinical decision-making in cancer medicine (Burrell et al., 2013). Thus, finding a “common” drug target or drug that works for all patients is challenging. An alternative approach is to develop a systematic classification for ccRCC and design effective therapeutic strategies for different patients' subtypes. Many studies have previously proposed some classification strategies based on the different genetic or transcriptomic characteristics and prognostic outcomes of patients (Brannon et al., 2010; Cancer Genome Atlas Research, 2013; Kosari et al., 2005; Takahashi et al., 2001). However, they failed to provide a clinically available biomarker for personalized classification/diagnosis and recommend targeted chemotherapy.

The problem is that some previously proposed biomarkers for ccRCC are based on a risk score summarized from the quantitative measurement of one or multiple signature genes (Fujita et al., 2012; Klatte et al., 2009). The application of risk score-based biomarkers needs a pre-setting threshold sensitive to the experimental batch effect (Guan et al., 2018). This brings the main barrier to translate the risk score-based biomarker to clinical practice (Winslow et al., 2012). In contrast, the biomarkers based on the within-sample relative expression orderings (REOs) of genes have been reported to be robust against batch effects (Guan et al., 2018), monotone data normalization (Eddy et al., 2010; Wang et al., 2013), and poor sample preparation (Chen et al., 2017; Cheng et al., 2017; Liu et al., 2017). Moreover, the prognostic value and classification performance have been widely validated in different cancer types (Ao et al., 2018; Chen et al., 2020; Guan et al., 2020; Lin et al., 2009; Qi et al., 2016). Thus, it is a promising alternative way to use the REOs-based methods to develop classification or prognostic biomarkers for ccRCC.

Systems biology-based methods are widely employed to identify drug targets and predict therapeutic agents based on drug repositioning (Altay et al., 2020; Lam et al., 2020, 2021; Mardinoglu et al., 2018; Ozcan et al., 2020). Genome-scale metabolic models (GEMs), one of the commonly used systems biology tools, have been used as a powerful tool to identify the potential drug targets inducing inhibition of tumor cell proliferation, which could be applied in drug repositioning (Mardinoglu and Nielsen, 2012; Zhang and Hua, 2015). The first step of the application of GEM is to create a subtype-specific model based on omics data of a subtype of samples, e.g., gene expression, by pruning a generic human GEM, which includes the comprehensive reactions, metabolites, and enzymes (Nilsson and Nielsen, 2017). Using this model, drug targets can be identified by essential gene analysis (Zhang et al., 2018). Besides, GEM can also be used to investigate whether it is toxic for a normal tissue cell after a gene knockout by computational modeling method (Agren et al., 2014; Uhlen et al., 2017). Kidney cancer has been considered a metabolic disease since it was characterized by various metabolic alterations associated with glucose metabolism, TCA cycle, fatty acid synthesis, and amino acid transport (Cancer Genome Atlas Research, 2013; Linehan and Ricketts, 2013; Linehan et al., 2019). Thus, it is suitable for ccRCC to use GEM analysis to identify drug targets.

**Figure 1:**
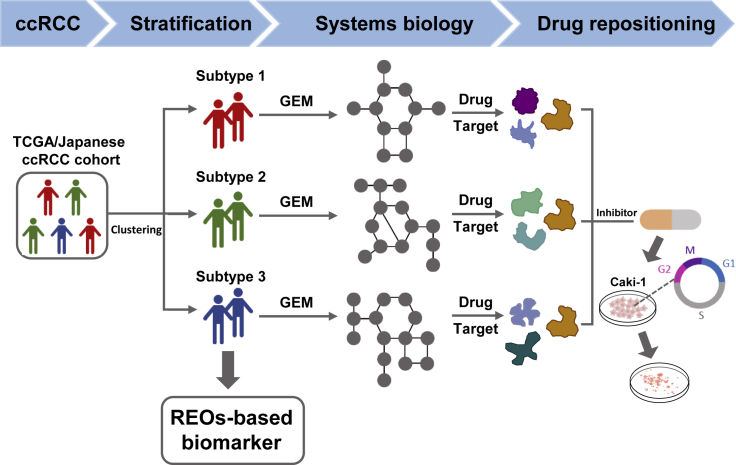
The flowchart for the whole study design including patient stratification, drug target identification, and drug repositioning

We performed a systems biology approach to stratify the patients with ccRCC into three subtypes with distinct mRNA expression patterns and clinical survival outcomes. Then, we developed a set of REO-based biomarkers that could precisely predict each subtype. Finally, we identified four potential drug targets using GEMs, repositioned mitotane for treatment of ccRCC by inhibiting one of the identified drug targets, *SOAT1* and validated its drug effect based on *in vitro* experiments. Figure 1 showed the whole study design.

## Results

### Identification of subtypes based on the different expression of mRNA

**Figure 2:**
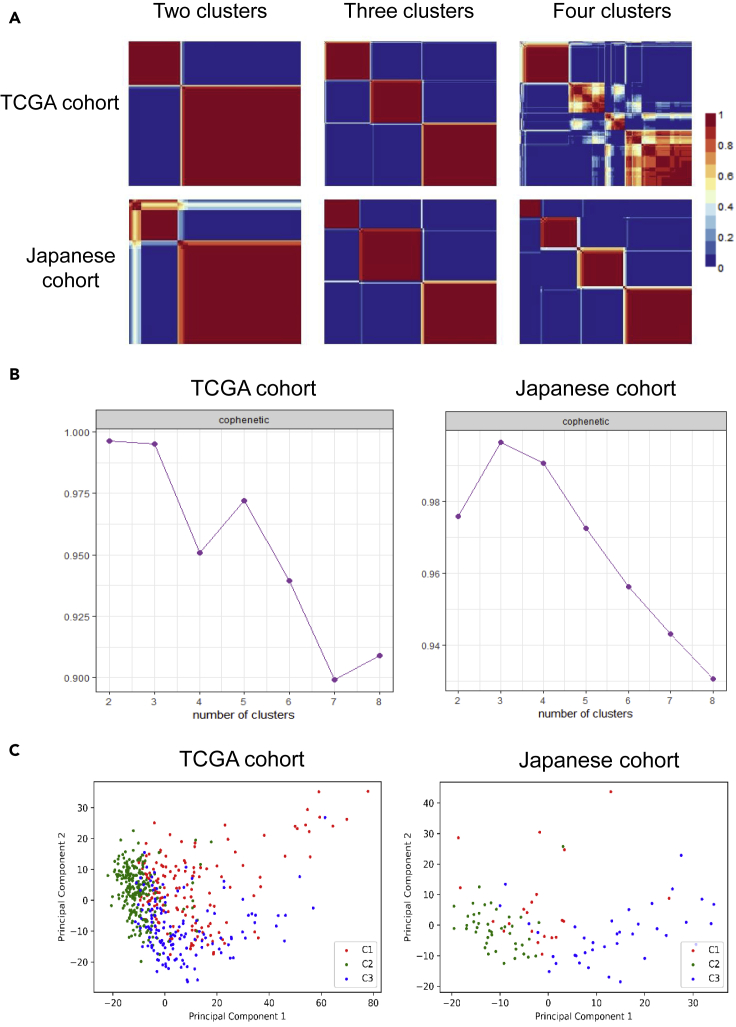
Molecular classification and distribution of samples of different subtypes (A) Reordered consensus matrices for ranks 2–4 (number of clusters) based on the overlapped 1264 genes with the highest MAD for samples from TCGA and Japanese cohorts. Dark blue corresponding to 0 means that the samples are never assigned to the same cluster. Dard red corresponding to 1 means that the samples are permanently assigned to the same cluster. The optimal cluster number is three since it shows the highest average of cophenectic coefficients in the two cohorts. In the middle plots, including three clusters, the subtype orders are 3, 1, and 2 for the TCGA cohort and 1, 3, and 2 for the Japanese cohort. (B) The distribution of *cophenetic* correlation coefficients when the samples were classified into different numbers of clusters. (C) PCA plot showing the distribution of samples from the three subtypes.

To develop a systematic classification, we first identified the top 1500 genes with the highest MAD values in the 530 and 100 samples of patients with ccRCC in TCGA and Japanese cohorts, respectively. We found that there were 1264 overlaps between the two cohorts. Based on the expression profiles of these 1264 genes, we employed an NMF clustering algorithm to stratify the samples into different sub-groups in TCGA and Japanese cohorts, respectively. The result showed an excellent classification when the samples were stratified into two or three clusters in both cohorts (Figure 2A). The optimal number of clusters was determined by the cophenetic correlation coefficient, which measured the stability of the identified clusters (Gaujoux and Seoighe, 2010). As shown in Figure 2B, we observed the highest average cophenetic correlation coefficient in the two cohorts when the samples were classified into three clusters. Thus, we determined an optimal number of three clusters and denoted them as subtypes 1, 2, and 3, respectively. Finally, we classified 156, 235, and 139 samples, and 21, 42, and 37 samples into subtype 1, 2, and 3 in TCGA and Japanese cohorts, respectively. Then we performed PCA analysis to visualize the distribution of all the samples. PCA plots showed good separation between subtype 2 and 3 in both cohorts, while subtype 1 was dispersed and mixed with the other two clusters (Figure 2C). In addition, we also performed PCA analysis for all samples merged from TCGA and Japanese cohorts after removing the batch effect. PCA plot even showed a more apparent separation between different subtypes, and the samples classified as the same subtype from the two cohorts were clustered together (Figure S1). These results suggested three distinct molecular subtypes with distinct mRNA expression in ccRCC, which has high confidence since the patients recruited in these two cohorts have different races and geographical differences.

### Clinical and molecular characteristics of subtypes

**Figure 3:**
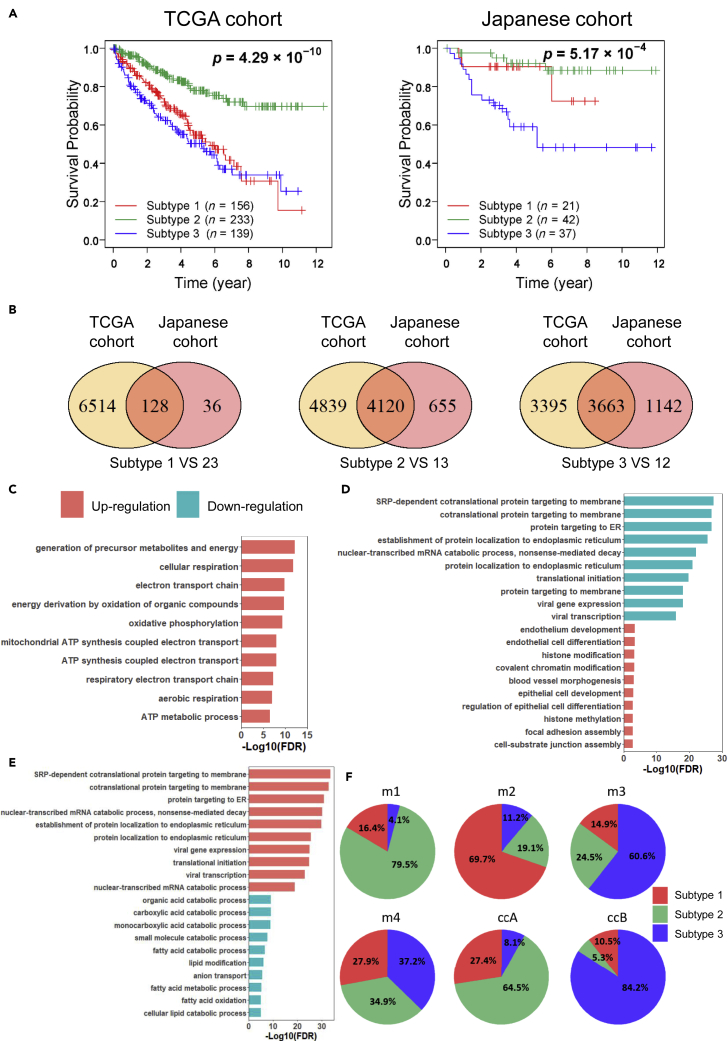
The survival outcomes and molecular characteristics for different subtypes (A) Kaplan-Meier plots of overall survival of the three subtypes in the TCGA and Japanese cohorts. (B) Venn diagrams showing the consistency of subtype-specific DEGs between TCGA and Japanese cohorts. (C) Top 10 most significantly GO pathways enriched with the overlapped subtypes-specific DEGs of subtype 1 between two cohorts. (D) Top 10 most significantly GO pathways enriched with the overlapped subtypes-specific DEGs of subtype 2 between two cohorts. (E) Top 10 most significantly GO pathways enriched with the overlapped subtypes-specific DEGs of subtype 3 between two cohorts. (F) Pie charts showing the intersection of the different classification systems for ccRCC. ‘m1’, ‘m2’, ‘m3’ and ‘m4’ indicate the molecular subtypes proposed by TCGA, and ‘ccA’ and ‘ccB’ are molecular subtypes reported by another previous study.

Survival analysis showed that the three subtypes of patients have significantly different overall survival in both the TCGA cohort (log rank p = 4.29 × 10^−10^) and the Japanese cohort (log rank p = 5.17 × 10^−4^) (Figure 3A). The patients in subtype 2 were associated with the best survival outcome with a 77.9% and 92.1% 5-year survival rate in TCGA and Japanese cohorts, respectively. The patients in subtype 3 were associated with the worst survival outcomes with a 50.3% and 59% 5-year survival rate in TCGA and Japanese cohorts, respectively. The prognostic outcomes of the patients in subtype 1 differed between cluster 2 and 3 in both cohorts.

To characterize different subtypes, we identified the DEGs between each subtype and the remaining two subtypes together in each cohort (Table S1). For example, we identified 6642 and 164 DEGs between the samples in subtype 1 and the remaining samples in the TCGA and Japanese cohorts, respectively (FDR<0.01, Figure 3B). The two lists of DEGs have a significant overlap (*k* = 132), and the concordance score of these overlapped genes between cohorts is 96.97% (hypergeometric distribution test, p = 2.63 × 10^−14^). Similarly, we found the number of overlapped DEGs identified in subtype 2 and subtype 3 are 4152 and 3689, the concordance score of these overlapped genes between cohorts is 99.23% and 99.3%, respectively (Figure 3B, hypergeometric distribution test, both p = 1.0 × 10^−16^). Then we performed GO enrichment analysis based on these consistently overlapped DEGs associated with each subtype, and we focused on the top 10 most significantly enriched GO pathways (FDR <0.01, Figures 3C–3E). We observed that the upregulated genes in subtype 1 were significantly enriched in the aerobic respiration, ATP synthesis and oxidative phosphorylation pathways, suggesting that the tumor cells of subtype 1 have a high activity of energy metabolism, which is necessary for cell viability (Figure 3C). However, the downregulated genes are not significantly enriched in any pathway. The upregulated genes in subtype 2 were significantly enriched in cell differentiation, histone modification, chromatin modification and focal adhesion assembly pathways (Figure 3D). It has been reported that the patients with cancer with good survival are associated with upregulation of cellular differentiation (Uhlen et al., 2017), which supports our result that the patients in subtype 2 show the best prognostic outcomes. The downregulated genes in subtype 2 are significantly enriched in translational initiation and protein localization to ER pathways, suggesting that transcription and translation are not activated in subtype 2, which also contribute to the good survival of patients in this subtype. The upregulated genes in subtype 3 were significantly enriched in the translational initiation and protein localization to ER pathways, which showed an opposite character to subtype 2 (Figure 3E).

Moreover, we found the viral transcription and viral gene expression pathways were upregulated in subtype 3 and downregulated in subtype 2, suggesting that these two subtypes' molecular characteristics may be associated with virus infection. It has been reported that the infection of hepatitis C virus, Epstein-Barr virus and human adenovirus viremia is associated with the development of kidney cancer (Hofmann et al., 2011; Kryst et al., 2020; Wu et al., 2021). The downregulated genes in subtype 3 were significantly enriched in fatty acid metabolism-related pathways such as fatty acid catabolic procession and fatty acid oxidation.

Further, we compared our classification with the previously reported TCGA (m1 to m4) (Cancer Genome Atlas Research, 2013) and ccA/ccB (Brannon et al., 2010) classification system (Figure 3F). In the TCGA cohort, 79.5% of TCGA m1 tumors were associated with our subtype 2 tumors, and the m1 group was also reported with the best prognostic outcomes in TCGA classification (Cancer Genome Atlas Research, 2013). Moreover, the m1 group was characterized by gene sets associated with chromatin remodeling processes, which showed a consistent result with the GO enrichment results for subtype 2. More than 60% of TCGA m2 and m3 tumors were associated with our subtype 1 and subtype 3 tumors, respectively, and m2 and m3 groups were also reported with poor prognosis in TCGA classification. Besides, TCGA m4 tumors comprised our subtype 1, 2 and 3 tumors with similar proportions, which showed a median survival in TCGA classification. In the Japanese cohort, 64.5% of ccA and 84.2% of ccB tumors were observed to be associated with our subtype 2 and 3 tumors, respectively. It has been reported that ccA cases have favorable survival (Brannon et al., 2010). These results demonstrated that the previous classification systems reinforce the three subtypes identified by our study.

### Development of the REO-based classification biomarker

To identify subtype-specific biomarkers that can be potentially used in clinical, we developed the REO-based biomarker by training the REOs of genes instead of absolute expression values of genes. Briefly, REO-based biomarkers used the subtype-specific gene pairs with opposite expression orders between a specific subtype of samples and the remaining samples as indicators and then optimized a minimum set of gene pairs as the final indicators for classification (See STAR Methods section). We merged the samples from TCGA and Japanese cohorts and randomly select 70% of samples as a training data set and the remaining 30% of samples as a validation data set. We identified 432, 73,652, and 21,978 subtype-specific gene pairs in subtypes 1, 2, and 3 in the training data set, respectively. We finally identified a set of REO-based biomarkers with a forward selection procedure consisting of 1, 21, and 19 gene pairs with an optimal F-score 0.8308 (precision = 0.8516, recall = 0.811), 0.9096 (precision = 0.9533, recall = 0.8697) and 0.9042 (precision = 0.9405, recall = 0.8706), respectively, which can precisely identify the patients in subtype 1, 2, and 3 (Table 1).

**Table 1:** The composition of classification biomarkers and voting rule

Subtype 1^a^		Subtype 2^a^		Subtype 3^a^	
Higher expression	Lower expression	Higher expression	Lower expression	Higher expression	Lower expression
*MT-ND5*	*RPS27*	*LRP2*	*BOLA3*	*BCL2L12*	*OPA3*
		*EHHADH*	*NDUFA4*	*RCC1*	*ACOX1*
		*RANBP2*	*TPST2*	*TARBP2*	*CLCN5*
		*LRP2*	*CCDC58*	*RCC1*	*MARK2*
		*MAP7*	*MMAB*	*ZNF581*	*SLC22A11*
		*FRYL*	*OSBPL3*	*NOB1*	*PCCA*
		*ITGA6*	*NDUFA4*	*ZNF581*	*CRY2*
		*GAREM1*	*MGME1*	*SEMA4B*	*RAPGEF2*
		*KIAA1671*	*GGCT*	*NLE1*	*CLCN5*
		*LRP2*	*TSR3*	*HSCB*	*GAREM1*
		*MAP7*	*C12orf45*	*IFT20*	*HIBCH*
		*LRP2*	*ATXN2L*	*S100A3*	*CDADC1*
		*ILK*	*MRPS24*	*FBXW9*	*C1orf210*
		*TOPORS*	*C12orf73*	*C19orf48*	*ANKIB1*
		*TLN2*	*MECR*	*STEAP3*	*SLC17A1*
		*EHHADH*	*BUD31*	*RPL39L*	*CLCN5*
		*DDAH1*	*TIMM13*	*PYM1*	*HIBCH*
		*LRP2*	*IFT22*	*USE1*	*PCCA*
		*EPHA4*	*NEURL2*	*SAT2*	*SLC22A11*
		*BPNT1*	*GGCT*		
		*EPHA4*	*PRELID3A*		

a For a given sample, if most of the gene pairs within a biomarker showed a higher expression for the former genes than the latter genes, this sample would be stratified into the specific subtypes.

For a given sample, if more than half of the gene pairs within a biomarker showed reversed REOs, this sample would be stratified into the specific subtypes. For example, if the expression of *MT-ND5* is higher than *RPS27* in a given sample, then this sample would be classified into subtype 1; otherwise, this sample does not belong to cluster 1 and should be predicted by the other two biomarkers (Table 1). We tested these biomarkers' performance in the validation dataset and found that the F-scores were 0.8537 (precision = 0.8846, recall = 0.8248), 0.815 (precision = 0.8242, recall = 0.8061), and 0.8591(precision = 0.913, recall = 0.8112) for the determination of subtype 1, 2, and 3, respectively. To further validate the prognostic value of these biomarkers, we applied the biomarkers in an independent European cohort (RECA-EU) with 91 KIRC samples. As a result, 27, 51, and 12 samples were stratified into subtype 1, 2, and 3, respectively. One sample was not classified into any subtype, which was removed in the following analysis. We observed that the patients of subtype 2 had significantly better survival outcome than subtype 3 (log rank p = 0.039) (Figure S2A). The patients in subtype 1 have a similar prognosis as the patients in subtype 2 (Figure S2B), consistent with what we observed in the Japanese cohort.

### Reconstruction of GEMs and drug target identification

**Figure 4:**
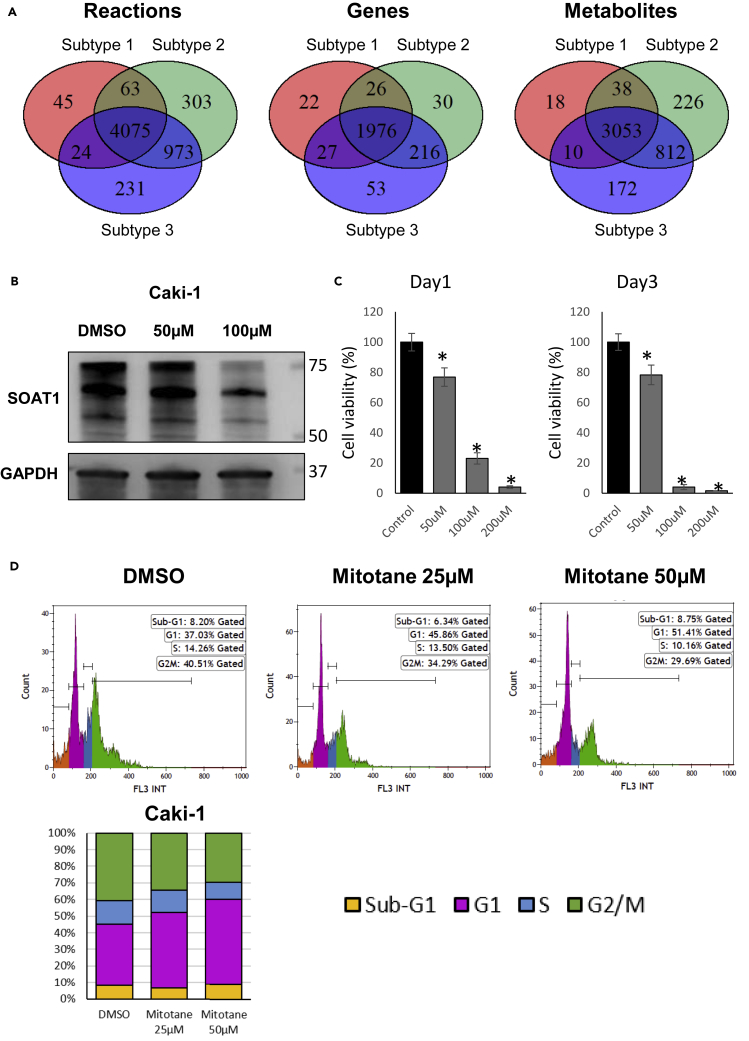
Metabolic model analysis and drug effect on ccRCC (A) Venn diagrams showing the overlaps of reactions, metabolites or genes between subtypes in the TCGA cohort. The reactions, metabolites, and genes are generated from reconstructed subtype-specific GEMs. (B) Western blot showing the protein level of SOAT1 was decreased by the treatment of mitotane in Caki-1. (C) Bar chart showing the cell viability was decreased by mitotane in Caki-1. ∗represents t test p < 0.05. Data are represented as mean ± SEM. (D) Flow cytometric analysis showing the cell cycle G2/M was arrested by mitotane in Caki-1.

We reconstructed the subtype-specific GEM by using the average gene expression values of each subtype in each cohort. We found 4207, 5414, and 5303 reactions, which comprised 2051, 2248, and 2272 genes and 3119, 4129, and 4047 metabolites in subtype 1, 2, and 3 in the TCGA cohort, respectively. Then we made a concordance analysis of reactions and metabolites between different clusters. As shown in Figure 4A, we found the three subtypes shared most of the reactions, genes and metabolites and also each subtype had its exclusive reactions, genes and metabolites. A similar result was observed in the Japanese cohort (Figure S3). The result provides the opportunity to identify common drug targets or subtype-specific drug targets by using GEM analysis.

Then we performed essentiality analysis in which GEMs are applied to identify essential genes and reactions whose knockout or blocking impacts critical biological functions of cell growth (Zhang et al., 2015). We set the objective function of the metabolic model to biomass maximization to identify the essential genes for tumor growth (Zhang et al., 2018). As a result, we found 42, 32, and 36 essential genes in subtype 1, 2, and 3 in the TCGA cohort and 52, 28, and 33 essential genes for these three clusters in the Japanese cohort. There were 37, 28, and 30 overlapped essential genes for each subtype between TCGA and Japanese cohorts, kept for further analysis (Table 2). Moreover, we performed an *in slico* toxicity test for each essential gene to test whether it is toxic in 32 major normal tissue cells after these gene knockouts (Agren et al., 2014; Uhlen et al., 2017) (See the STAR Methods section). We hypothesized that if a gene is an essential gene in ccRCC cell and its knockout is not toxic for normal tissue cells, it could be treated as a potential drug target. Finally, after removing the genes whose knockout was toxic in major human normal tissue cells, we filtered out four genes, *SOAT1*, *CRLS1,* and *ACACB*, which are essential for all three subtypes of ccRCC, showing the potential to be used as drug targets for the majority of patients with ccRCC regardless of specific molecular subtype, as well as *GPD2* which is essential for subtype 1 specifically.

**Table 2:** Candidate essential gene list for each subtype.

Gene symbol	Essential in subtype 1	Essential in subtype 2	Essential in subtype 3
SOAT1	1^a^	1	1
CRLS1	1	1	1
ACACB	1	1	1
CYP51A1	1	1	1
IDI1	1	1	1
FDFT1	1	1	1
PGS1	1	1	1
CRAT	1	1	1
TECR	1	1	1
CDIPT	1	1	1
SQLE	1	1	1
SC5D	1	1	1
MVK	1	1	1
HMGCR	1	1	1
DHCR24	1	1	1
HSD17B7	1	1	1
CMPK2	1	1	1
GUK1	1	1	1
EBP	1	1	1
NSDHL	1	1	1
HSD17B12	1	1	1
LSS	1	1	1
PMVK	1	1	1
MVD	1	1	1
DTYMK	1	1	1
DHCR7	1	1	1
SLC22A5	1	1	1
GPD2	1	0^b^	0
PISD	1	0	1
ACAA1	1	0	0
ABCD1	1	0	0
EHHADH	1	0	0
ACAT2	1	0	0
CAT	1	0	0
DEGS1	1	0	0
PCYT2	1	0	0
ACADSB	1	0	0
SGPL1	0	1	0
PTDSS1	0	0	1
PCYT1A	0	0	1

a 1 means this gene is essential in this subtype.

b 0 means this gene is not essential in this subtype.

Next, we would like to experimentally test the predicted drug targets for each subtype using *in vitro* model. As we could not classify cell line into a specific subtype, we decided to evaluate one of the common drugs we predicted as a proof of concept. In this case, we selected *SOAT1*, an enzyme catalyzing the formation of fatty acid-cholesterol esters, which was indicated as a possible drug target in adrenocortical carcinoma (Sbiera et al., 2015) and glioblastoma (Geng et al., 2016) for our validation. Mitotane, an inhibitor of *SOAT1* (Sbiera et al., 2015), is an FDA-approved small molecule drug commonly used to treat adrenocortical carcinoma (Paragliola et al., 2018). To explore the drug effect of mitotane on ccRCC, we treated Caki-1, a ccRCC cell line, with mitotane. As shown in Figure 4B, we observed that the protein level of *SOAT1* was significantly decreased by mitotane treatment compared to the negative control, which suggested that the drug successfully targets *SOAT1* as expected. Cell viability was significantly reduced with increasing concentrations of mitotane (Figure 4C). Further, we observed the drug inhibited cell growth through G2/M cell cycle arrest in Caki-1 (Figure 4D). These results suggested that mitotane is a promising drug for the treatment of ccRCC in clinical practice.

## Discussion

In this study, we used a systems biology approach to identify three different molecular subtypes with distinct molecular characteristics and different survival outcomes in ccRCC. Two of these subtypes, subtype 2 and 3 correspondingly associated with the best and worst prognostic outcomes, respectively. These subtypes have potentially opposite characteristics since pathways related to translational initiation, protein targeting and localization, viral transcription and viral gene expression pathways were downregulated in subtype 2 but upregulated in subtype 3. This classification provided a new insight that these two subtypes may be associated with virus infection. Moreover, the high grade of tumor cell differentiation potentially explained the good survival outcomes of patients in subtype 2. The tumors of subtype 1 were characterized by an active energy metabolism, which was a middle subtype between subtype 2 and 3 in terms of survival outcomes. We observed that the survival outcome of subtype 1 among the three cohorts (TCGA, Japanese and European cohorts) performed slightly different, since survival outcome is a complex phenotype not only decided by molecular profiles but also decided by races, geography, eating habits, and treatment strategies (Stafford et al., 2008; Zeng et al., 2015).

Next, we developed a set of subtype-specific biomarkers based on the merged training data set to include biological and cultural background differences in the REOs-based biomarker during training since the patients from TCGA, and Japanese cohorts had quite different races. The result showed that the REOs of our biomarkers could robustly stratify the patients into different subtypes in the validation data set and also an independent European ccRCC cohort. Although we used RNA-seq data for biomarker application in this study, a more convenient and cheaper technology, RT-PCR, could be a good alternative since we only need to measure the REOs of gene pairs involved in the biomarker. In previous studies, most classification biomarkers are based on the risk score summarized from absolute expression values of signature genes. Their application compares the risk score calculated from the gene expression of a given sample with preset risk score thresholds. There are two most famous signatures which are already used in commercial. AlloMap, consisting of 20 genes, predicts the risk of acute cellular rejection in heart transplant recipients (Starling et al., 2006). Oncotype DX, consisting of 21 genes, is used to estimate the risk of distant recurrence in tamoxifen-treated patients with node-negative, estrogen-receptor-positive breast (Paik et al., 2004). Both signatures employ RT-PCR to detect the expression levels of the signature genes and then calculate the risk score. Because of batch effect and platform differences, the generated risk score cannot be directly compared with the preset thresholds (Qi et al., 2016). Considering this, the collected samples must be measured in specified laboratories with strict quality control and uniform normalization, which significantly limits the wide application of these biomarkers. In contrast, REOs-based biomarkers are relatively insensitive to experimental batch effect and invariant to monotone data transformation (Guan et al., 2018) and they are very promising in clinical practice. Moreover, the REO of two genes in a gene pair is easy to measure by RT-PCR with a proper operation. These advantages facilitate the use of REO-based biomarker in personalized medicine.

Based on metabolic modeling analysis, we identified four target genes, namely *SOAT1*, *CRLS1*, *ACACB,* and *GPD2*, whose inhibition can block the growth of ccRCC tumor cells with relatively low toxicity to normal tissue cells. It has been reported that *SOAT1* is a potential drug target in adrenocortical carcinoma (Sbiera et al., 2015) and glioblastoma (Geng et al., 2016). *CRLS1* encodes an enzyme that catalyzes the synthesis of cardiolipin, which is a phospholipid component of mitochondrial membranes and critical for mitochondrial function. As a potential therapeutic target, silencing of *CRLS1* inhibited cell growth in liver cancer (Bidkhori et al., 2018). *ACACB* encodes a rate-limiting enzyme of fatty acid synthesis. Fatty acids are necessary for tumor cells to synthesize membranes and signaling molecules. It has been reported that aberrant expression of *ACACB* increases the risk in different cancer types (Currie et al., 2013). *GPD2* encodes an enzyme localized in the inner mitochondrial membrane, which catalyzes the conversion of glycerol-3-phosphate to dihydroxyacetone phosphate. The upregulation of this gene increased the glycolysis in different cancer types (Lu et al., 2020; Mikeli et al., 2020; Wu et al., 2020).

We observed that three of the four targets, including *SOAT1*, *CRLS1*, *ACACB,* are the common drug targets that are effective for all subtypes. This was a surprising finding in this study since it is challenging to find a shared drug target, effective for all subtypes, due to the high inter-tumor or intra-tumor heterogeneity (Burrell et al., 2013). Hence, we validated the inhibitory effect of the common targets in a general ccRCC cell line model without specific subtype determination. Since there is a known inhibitor of *SOAT1*, mitotane, we finally selected and validated the anti-cancer effect of *SOAT1* in a widely used ccRCC cell line Caki-1. This could serve as a proof of concept validation to show that the target genes we identified are promising and could be potentially used for ccRCC treatment.

In conclusion, we identified three molecular subtypes in ccRCC and proposed a set of clinically promising REOs-based classification biomarkers for subtype diagnosis at individual level. We also successfully validated our findings in three different independent cohorts. In addition, we identified specific gene targets for the treatment of the subtype(s), and validated one of the common targets, *SOAT1,* using an *in vitro* model. Therefore, this study provides new insight into ccRCC molecular subtypes and proposes practical strategies for personalized diagnosis and precision medicine on subtype level treatment.

### Limitations of the study

Although we proposed subtype-specific drug target such as *GPD2* in this study, it was not possible to validate its inhibition effects in a general cell line model because there is no way to determine the subtype category using cell lines. An ideal validation study should be performed using subtype-specific patient-derived cell lines or their xenograft models. Besides GPD2, we also reported some other promising subtype-specific gene targets such as *ACAA1*, *ABCD1,* and *EHHADH,* and so on, for subtype 1, *SGPL1* for subtype 2 and *PTDSS1* and *PCYT1A* for subtype 3 as presented in Table 2. Some of these targets may be toxic to some other tissues based on *in silico* simulations. However, it is still worthwhile to evaluate their drugability using *in vitro* and *in vivo* models. Thus, it will be interesting to evaluate these targets in future studies by including additional patients with ccRCC and generating subtype-specific patient-derived cell lines or xenograft models.

## STAR★Methods

### Key resources table

**Table:** 

REAGENT or RESOURCE	SOURCE	IDENTIFIER
**Antibodies**		

Anti-SOAT1/ACAT1 antibody	Abcam	Cat# ab39327
GAPDH antibody	Santa Cruz Biotechnology, Inc.	Cat# sc-47724
Secondary antibody, goat Anti-Rabbit HRP	Abcam	Cat# ab205718
goat anti-mouse IgG-HRP	Santa Cruz Biotechnology, Inc.	Cat# sc-2005

**Chemicals, peptides, and recombinant proteins**		

Mitotane	Sigma-Aldrich, Saint Louis, MO, USA	SML1885

**Deposited data**		

TCGA ccRCCC samples	Tatlow, P.J., and Piccolo, S.R. (2016). A cloud-based workflow to quantify transcript-expression levels in public cancer compendia. Sci Rep *6*, 39259. 10.1038/srep39259.	https://osf.io/gqrz9 (TCGA_KIRC_tpm.tsv.gz;TCGA_KIRC_counts.tsv.gz)
Japanese ccRCC samples	European Genome-phenome Archive	EGAS00001000509 (https://ega-archive.org/studies/EGAS00001000509?order=samples&sort=asc)
European ccRCC samples	ICGC	RECA-EU (https://dcc.icgc.org/releases/current/Projects/RECA-EU)

**Experimental models: cell lines**		

Caki-1	CLS Cell Lines Service GmbH, Eppelheim, Germany	RRID:CVCL_0234

**Software and algorithms**		

R language version 4.0.3	formerly AT&T, now Lucent Technologies	https://www.r-project.org/
Matlab language version R2017b	Mathwork	https://ch.mathworks.com/products/matlab.html
Zenodo	CERN	10.5281/zenodo.4906949

### Resource availability

#### Lead contact

Further information and requests for resources and reagents should be directed to and will be fulfilled by the lead contract, Adil Mardinoglu (adilm@scilifelab.se)

#### Materials availability

This study did not generate new unique reagents.

#### Data and code availability

This paper analyzes existing, publicly available data. The accession numbers or URL for the datasets are listed in the key resources table.

All original code has been deposited at Zenodo and is publicly available as of the data of publication. DOI is listed in the key resources table.

Any additional information required to reanalyze the data reported in this paper is available from the lead contract upon result.

### Experimental model and subject details

Human ccRCC cell line Caki-1 was purchased in CLS (RRID: CVCL_0234, CLS Cell Lines Service GmbH, Eppelheim, Germany), which was derived from a male ccRCC patient. Cells were cultured with proliferation media, McCoy’s 5A Medium (M9309, Sigma-Aldrich, Saint Louis, MO, USA) with 10% FBS and 1% Penicillin/Streptomycin supplemented media, 37°C.

### Method details

#### MTT assay

Mitotane (SML1885, Sigma-Aldrich, Saint Louis, MO, USA) was dissolved in DMSO. Cell viability was measured with an MTT assay. Caki-1 cells were seeded into a 96-well plate at 5,000 cells per well triplicated. Day after seeding, mitotane was treated with media change at the proper concentration. 10x MTT solution (5mg/ml) was dissolved into proliferation media to 1x concentration. Media was changed into 1x MTT solution and incubated for 2 hours. Formazan stained cells were dissolved with 80μl of DMSO and measured with a microplate reader (Hidex Sense Beta Plus) at O.D 570nm.

#### Western blots

The whole cell lysate was prepared with CelLytic M (C2978, Sigma-Aldrich, Saint Louis, MO, USA) buffer. SDS PAGE was performed with 30μg of lysate into Mini-PROTEAN® TGX™ Precast Gels (Bio-Rad, CA, USA) and transferred using Trans-Blot® Turbo™ Transfer System (Bio-Rad, CA, USA). Anti-SOAT1/ACAT1 antibody (ab39327, Abcam), GAPDH antibody (sc47724, Santa Cruz Biotechnology, Inc.) were blotted overnight. Secondary antibody, goat Anti-Rabbit HRP (ab205718), and goat anti-mouse IgG-HRP (sc2005, Santa Cruz Biotechnology, Inc.) were blotted for one hour. Protein band was detected with ImageQuant^TM^LAS 500 (29-0050-63, GE). All antibodies were diluted at a 1:10000 concentration.

#### Flow cytometric analysis (FACs)

Propidium Iodide (P3566, Sigma-Aldrich) staining was performed for cell cycle analysis. Two days mitotane-treated Caki-1 cells were trypsinized. Centrifuged cells were re-suspended in 1ml PBS. Cells were fixed by adding 2.5 ml ethanol and incubated for 15 minutes on ice. After centrifugation, cells were re-suspended with 500μl of PI staining solution consist of 10 μg/ml propidium iodide, 20 μg/ml RNase A (12091021, ThermoFisher), 0.05% triton X-100 in PBS for 20 minutes at dark and room temperature. 3 mL PBS was added to PI stained cells and centrifuged again. 1 ml PBS re-suspended cells analyzed by FACs (BECKMAN COULTER NAVIOS^TM^). PI stained cells were gated at 10,000 cells and 3,000 cells that less than 300 FS INT were used for cell cycle analysis. Data was produced with Kazula Analysis Version 2.1.

### Quantification and statistical analysis

#### Data and preprocessing

Global transcript-expression profiles (TPM and count values) of 530 TCGA ccRCC samples were downloaded from https://osf.io/gqrz9 (Tatlow and Piccolo, 2016). We extracted the tumor samples with sample and vial identifiers of BRC patient barcode ‘01A′, which represented primary solid tumor tissue from the first vial. The mRNA expression was quantified using Kallisto (Bray et al., 2016) based on the GENCODE reference transcriptome (version 24) (Ensembl 83 (GRCh38.P5)). We downloaded the clinical information of TCGA samples by using the R package TCGAbiolinks (Colaprico et al., 2016). The whole-exome sequences data of 100 KIRC samples of patients from the Japanese cohort (Sato et al., 2013) were downloaded from European Genome-phenome Archive (accession number: EGAS00001000509). We used BEDTools (Quinlan and Hall, 2010) to convert BAM to FASTQ file. Kallisto was used for estimating the count and TPM values of transcripts based on the same reference transcriptome of TCGA data. The sum value of the multiple transcripts of a gene was used as the expression value of this gene. The gene-level expression profiles (RPKM values) of 91 European ccRCC samples were downloaded from https://dcc.icgc.org/releases/current/Projects/RECA-EU. The genes with average TPM/RPKM values >1 were analyzed.

#### Clustering analysis

We extracted the top 1500 genes with the highest mean absolute deviation (MAD) of gene expression in TCGA and Japanese cohorts, respectively. Then 1264 overlapped genes between the two cohorts were further used for clustering. The Standard NMF algorithm proposed by Brunet (Brunet et al., 2004) was used for sample clustering based on the expression values of the 1264 genes by R package NFM which is based on non-negative matrix factorization (NMF) algorithm. To find stable clusters, 200 iterations of NMF were run for each possible number of clusters between two to eight. The optimal number of clusters was determined based on the cophenetic correlation coefficient, which is a measurement of clusters' stability (Brunet et al., 2004; Gaujoux and Seoighe, 2010). Principal component analysis (PCA) was used to visualize the distribution of samples based on R function prcomp.

#### Differential expression analysis

The function ‘removeBatchEffect’ from the limma package was used to remove batch effect for merged TPM expression profiles (Ritchie et al., 2015). DESeq2 (Love et al., 2014) was used to identify differentially expressed genes (DEGs) between two groups. The lowly expressed genes with average TPM≤1 were removed, and the raw count values of the remaining genes were used as the input of DESeq2. Gene ontology (GO) enrichment was performed based on the R package ClusterProfiler (Yu et al., 2012). This tool uses the hypergeometric distribution to estimate whether a list of DEGs is significantly enriched in each GO pathway. False discovery rate (FDR) was adjusted by the Benjamini-Hochberg (BH) method. FDR<0.01 was used to identify significant DEGs and enriched pathways.

#### Concordance analysis of DEGs

If two lists of DEGs, list 1 with *L*_*1*_ genes and list 2 with *L*_*2*_ genes, have *k* overlapping genes, among which *s* genes shows the same dysregulated directions (up or down-regulation) in the two DEGs lists, the probability of observing at least *s* consistent genes by chance can be estimated based on the following cumulative hypergeometric distribution model:

$$P=1-\sum_{i=0}^{s-1}\frac{\left(\begin{array}{l} L_{2}\\ i \end{array}\right)\left(\begin{array}{l} L-L_{2}\\ L_{1}-i \end{array}\right)}{\left(\begin{array}{l} L\\ L_{1} \end{array}\right)}$$

Where, *L* represents the number of the background genes commonly measured in the datasets from which the DEGs are extracted. The two DEG lists were considered to be significantly overlapped if p  <  0.05. The concordance score *s/k* is used to represent the consistency of DEGs between the two lists. The score ranges from 0 to 1, and the higher concordance score indicates the better consistency of two lists of DEGs.

#### Development of the REOs-based biomarker

In a given sample, the relative expression ordering (REO) of every two genes (*i* and *j*) is denoted as either G_i_ > G_j_ or G_i_ < G_j_ exclusively, where G_i_ and G_j_ represent the expression values (TPM) of gene *i* and *j*, respectively. For a given gene pair (G_i_ and G_j_), we used a binomial test to evaluate whether the frequency of a specific REO pattern (G_i_ > G_j_ or G_i_ < G_j_) was significantly stable in a cluster of samples as follows:

$$P=1-\sum_{i=0}^{k-1}\left(\begin{array}{l} n\\ i \end{array}\right)p_{0}^{i}{\left(1-p_{0}\right)}^{n-i}$$

Where n denotes the total number of samples in the cluster, k denotes the number of samples with a certain REO pattern (G_i_ > G_j_ or G_i_ < G_j_) and$p_{0}$($p_{0}$=0.5) is the probability of observing a certain REO pattern in a sample by chance. The p values are adjusted based on the BH method. FDR < 1. × 10^−7^ was used to detect the significantly stable gene pairs.

The subtype-specific gene pair shows a significantly stable REO in one subtype (e.g., subtype 1). However, its REO is changed to a reversed pattern and still keeps stable in the remaining samples (e.g. cluster 2 plus cluster 3), which was used to train the subtype-specific biomarker. In total, we found 432, 73652 and 21978 subtype-specific gene pairs for cluster 1, 2 and 3 in the training dataset. We predicted whether a given sample belongs to the specific subtype based on the REO of each subtype-specific gene pair. Here, precision is defined as the ratio of correctly identified subtype-specific samples to all subtype-specific samples. The recall is defined as the ratio of correctly determined non-subtype-specific samples to all non-subtype-specific samples. Then, from these subtype-specific gene pairs, we performed a forward selection procedure in the training dataset to search a subset of gene pairs that achieved the highest F-score value, a harmonic mean of precision and recall, which is calculated as follows:

$$F-score=2\cdot \frac{Precision\cdot Recall}{Precision+Recall}$$

Using the subtype-specific biomarker of subtype 1 as an example, among the 432 subtype-specific gene pairs, we selected the gene pair with the highest F-score as a seed. Next, we added another gene pair to the biomarker until the F-score did not increase. The gene pair whose join cannot improve F-score is not added during selection. The classification rule is that a sample is classified into a specific subtype if most of the REOs within the biomarker vote for this subtype.

#### GEMs analysis

We reconstructed the subtype-specific GEMs based on the average expression values of the genes in each cluster in the TCGA and Japanese cohorts, respectively. The expression of genes was divided into four levels, no expression with TPM < 1, low expression with 1≤TPM<10, median expression with 10≤TPM<50 and high expression with TPM≥50. The iCancerCore model (Uhlen et al., 2017) was used as a template model for GEM reconstruction through Integrative Network Inference for Tissues algorithm (tINIT) (Agren et al., 2014) and the Mosek solver (version 7) in the RAVEN Toolbox (Agren et al., 2013). We used the ESS tool to extract essential genes that are necessary for tumor cell growth (Zhang et al., 2018). The threshold of cell growth rate was set to 0.05. After we generated the essential gene list, an in silico toxicity test was performed in 32 available normal tissue models (Uhlen et al., 2017), reconstructed with the tINIT algorithm, to assess if the knockout of these genes is toxic for normal cells. Our previous study defined 56 mandatory metabolic tasks categorized as energy and redox, internal conversions, substrate utilization and biosynthesis of products (Agren et al., 2014), whose balance is necessary for normal cells to execute their basic metabolic function. During the toxicity test, each of the putative gene targets is computationally knocked out in the 32 normal tissue models to test if their silence interrupts the mandatory metabolic tasks. The gene targets which do not interrupt any of the mandatory metabolic task in any of the normal tissue model are considered to be less toxic (Agren et al., 2014). These genes will be kept in the candidate drug target list.

## Supplemental information

Document S1. Figures S1–S3

Table S1. The DEGs between each subtype and the remaining two subtypes together in each cohort, related to Figure 3B
