# Supplementary material for: Stratification of patients with clear cell renal cell carcinoma to facilitate drug repositioning
Source: iScience. 2021 Jun 12;24(7):102722. doi: 10.1016/j.isci.2021.102722 (PMC8253978; doi:10.1016/j.isci.2021.102722)

iScience, Volume *24*

Supplemental information

Stratification of patients with clear cell renal cell carcinoma to facilitate drug repositioning

Xiangyu Li, Woonghee Kim, Kajetan Juszczak, Muhammad Arif, Yusuke Sato, Haruki Kume, Seishi Ogawa, Hasan Turkez, Jan Boren, Jens Nielsen, Mathias Uhlen, Cheng Zhang, and Adil Mardinoglu

Figure S1. PCA plot showing the distribution of samples from TCGA and Japanese cohort, related to Figure 2C. ComBat-seq method was used to remove the batch effect of merged expression profiles before PCA analysis.

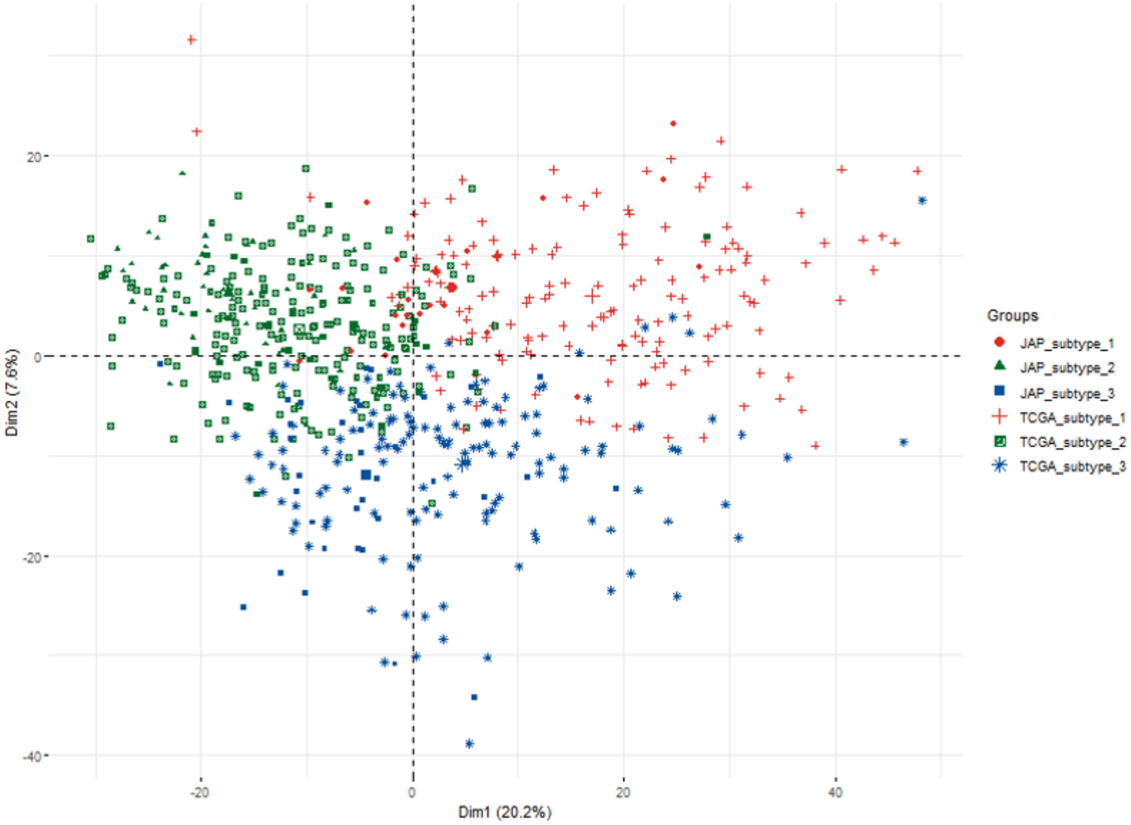

Figure S2. Kaplan-Meier plots of overall survival of the three subtypes in an independent European ccRCC cohort, related to Figure 3A. (A) Kaplan-Meier plot for cluster 2 and 3. (B) Kaplan-Meier plot for cluster 1, 2 and 3.

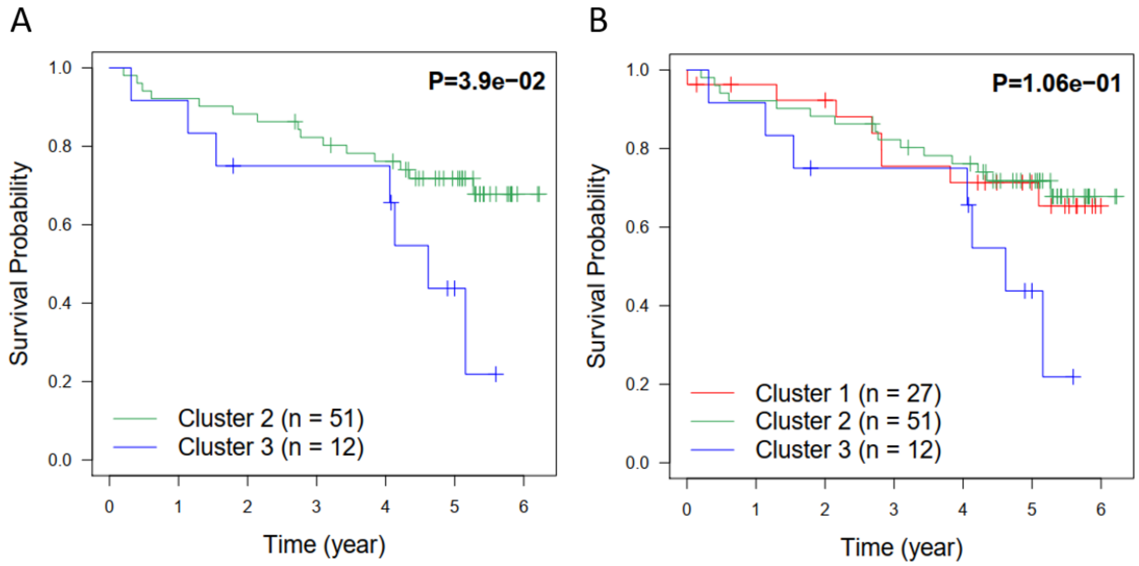

Figure S3. Venn diagrams showing the overlaps of reactions, metabolites or genes between subtypes in the Japanese cohort, related to Figure 4A. The reactions, metabolites and genes are generated from reconstructed subtype-specific GEMs.

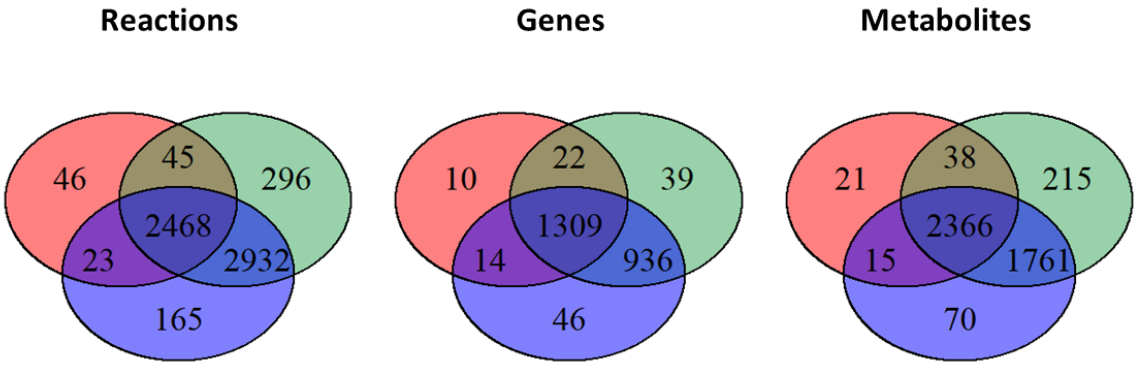

Supplement: Document S1. Figures S1–S3 [file mmc1.pdf]
